# EF-Tu dynamics during pre-translocation complex formation: EF-Tu·GDP exits the ribosome via two different pathways

**DOI:** 10.1093/nar/gkv856

**Published:** 2015-09-03

**Authors:** Wei Liu, Chunlai Chen, Darius Kavaliauskas, Charlotte R. Knudsen, Yale E. Goldman, Barry S. Cooperman

**Affiliations:** 1 Department of Chemistry, University of Pennsylvania, Philadelphia, PA 19104, USA; 2 Pennsylvania Muscle Institute, Perelman School of Medicine, University of Pennsylvania, Philadelphia, PA 19104, USA; 3 Department of Molecular Biology and Genetics and Interdisciplinary Nanoscience Center (iNANO), Aarhus University, DK-8000 Aarhus C, Denmark

## Abstract

The G-protein EF-Tu, which undergoes a major conformational change when EF-Tu·GTP is converted to EF-Tu·GDP, forms part of an aminoacyl(aa)-tRNA·EF-Tu·GTP ternary complex (TC) that accelerates the binding of aa-tRNA to the ribosome during peptide elongation. Such binding, placing a portion of EF-Tu in contact with the GTPase Associated Center (GAC), is followed by GTP hydrolysis and P_i_ release, and results in formation of a pretranslocation (PRE) complex. Although tRNA movement through the ribosome during PRE complex formation has been extensively studied, comparatively little is known about the dynamics of EF-Tu interaction with either the ribosome or aa-tRNA. Here we examine these dynamics, utilizing ensemble and single molecule assays employing fluorescent labeled derivatives of EF-Tu, tRNA, and the ribosome to measure changes in either FRET efficiency or fluorescence intensity during PRE complex formation. Our results indicate that ribosome-bound EF-Tu separates from the GAC prior to its full separation from aa-tRNA, and suggest that EF-Tu·GDP dissociates from the ribosome by two different pathways. These pathways correspond to either reversible EF-Tu·GDP dissociation from the ribosome prior to the major conformational change in EF-Tu that follows GTP hydrolysis, or irreversible dissociation after or concomitant with this conformational change.

## INTRODUCTION

EF-Tu is, along with EF-G, one of two G-protein factors that are required for nascent polypeptide elongation by the prokaryotic 70S ribosome (1). In the first elongation cycle, EF-Tu binds as part of an aminoacyl(aa)-tRNA·EF-Tu·GTP ternary complex (TC) to the 70S initiation complex (70SIC), which contains initiator fMet-tRNA^fMet^ bound in the P-site. The TC initially binds to the A/T site of the ribosome, placing a portion of EF-Tu in contact with the GTPase Associated Center (GAC) of the 50S subunit (2). Formation of base pairs between the anticodon loop of aa-tRNA and the cognate codon triplet in mRNA activates EF-Tu GTPase activity. This is followed by GTP hydrolysis, P_i_ release, aa-tRNA accommodation into the A-site, peptide bond formation, and, finally, EF-Tu·GDP dissociation from the ribosome, resulting in pretranslocation (PRE) complex formation. EF-G·GTP catalysis of the translocation of fMet-aa-tRNA and tRNA^fMet^ from the A- and P-sites to the P- and E-sites, respectively, along with their bound mRNA codons, completes the first elongation cycle by the formation of the posttranslocation (POST) complex.

Although the movement of tRNA through the ribosome during PRE complex formation has been extensively studied by kinetic, structural and modeling studies (3–7), less is known about the dynamics of EF-Tu interaction with either the ribosome or aa-tRNA during this process. For example, it is unclear at what point the contacts between EF-Tu and aa-tRNA or the GAC are broken. Also unclear is the timing of the major conformational change that EF-Tu undergoes when the EF-Tu·GTP conformation, as modeled by replacing GTP with GDPNP in either the binary complex EF-Tu·GDPNP (8,9) or the TC, EF-Tu·GDPNP·Phe-tRNA^Phe^ (10), is converted to the EF-Tu·GDP conformation (11,12), although it has been suggested that it occurs concomitantly with P_i_ release (13,14).

Recently, we described the development of two fluorescence resonance energy transfer (FRET) assays monitoring, during PRE complex formation, the proximity of EF-Tu to the D-loop of aa-tRNA (EF-Tu:tRNA assay) and to the C-terminal domain (residue 87) of ribosomal protein L11 (15), which lies within the GAC (EF-Tu:L11 assay). These assays utilize functionally active fluorescent and fluorescence quencher derivatives of EF-Tu labeled at position 348 (Supplementary Figure S1). Here we use these assays, in both ensemble and single molecule formats, together with other assays measuring TC interaction with 70SIC, to address the issues posed above. Our results indicate that ribosome-bound EF-Tu separates from L11 prior to its full separation from aa-tRNA, and that EF-Tu·GDP dissociates from the ribosome by two different pathways. They further suggest that these pathways correspond, respectively, to EF-Tu·GDP dissociation from the ribosome occurring prior to or accompanying the major conformational change in EF-Tu.

## MATERIALS AND METHODS

### Labeled protein synthesis components

70SIC^Cy3^ and 70SIC^Cy5^ (16), EF-Tu^QSY9^, EF-Tu^Cy3^, EF-Tu^Cy5^ and EF-Tu^AV-Cy5^ (15), Phe-tRNA^Phe^(Cy3) and Phe-tRNA^Phe^(Cy5) (17), labeled TCs (15) and fMet-tRNA^fMet^(prf) and coumarin-labeled phosphate binding protein (C-PBP) (18) were prepared using described procedures.

### Labeled component nomenclature

Labeled initiation complexes, denoted 70SIC^Cy3^ and 70SIC^Cy5^, contained protein L11 labeled at position 87 with either Cy3 or Cy5, respectively. EF-Tu derivatives prepared from the single-mutation variant of EF-Tu, E348C-EF-Tu, are denoted EF-Tu^QSY9^ (QSY9 is a dark fluorescence quencher), EF-Tu^Cy3^ and EF-Tu^Cy5^ (15). A fourth derivative, denoted EF-Tu^AV-Cy5^, was prepared from the triple-mutation variant C137A/C255V/E348C-EF-Tu (15). tRNAs labeled at dihydrouridine positions in the D-loop are denoted Phe-tRNA^Phe^(Cy3), Phe-tRNA^Phe^(Cy5) and fMet-tRNA^fMet^ (prf), where prf is an abbreviation for proflavin. Labeled TCs were prepared from labeled EF-Tu derivatives and either unlabeled Phe-tRNA^Phe^, Phe-tRNA^Phe^(Cy3), or Phe-tRNA^Phe^(Cy5) and named as indicated in Table 1.

**Table 1.** Labeled ternary complexes (TCs)

TC name	Labeled EF-Tu variant	Phe-tRNA^Phe^
TC^QSY9^	E348C-EF-Tu^QSY9^	Phe-tRNA^Phe^
TC^QSY9/Cy3^	E348C-EF-Tu^QSY9^	Phe-tRNA^Phe^(Cy3)
TC^Cy3^	E348C-EF-Tu^Cy3^	Phe-tRNA^Phe^
TC^Cy3/Cy5^	E348C-EF-Tu^Cy3^	Phe-tRNA^Phe^(Cy5)
TC^Cy5^	E348C-EF-Tu^Cy5^	Phe-tRNA^Phe^
TC^Cy5/Cy3^	E348C-EF-Tu^Cy5^	Phe-tRNA^Phe^(Cy3)
TC^AV-Cy5^	E348C-EF-Tu^AV-Cy5^	Phe-tRNA^Phe^
TC^AV-Cy5/Cy3^	E348C-EF-Tu^AV-Cy5^	Phe-tRNA^Phe^(Cy3)

### Complex preparation

All complexes were prepared in buffer A (50 mM Tris-HCl [pH7.5], 70 mM NH_4_Cl, 30 mM KCl, 7 mM MgCl_2_ and 1 mM DTT) at 37°C as described (15). 70SICs were programmed with mRNA 022 (19). For those experiments using fMet-tRNA^fMet^(prf), C-PBP or GDPNP, further purification of 70SIC was performed by ultracentrifugation through a 1.1 M sucrose cushion in buffer A (SORVALL S120-AT2 rotor, 110K rpm, 40 min, 4°C).

### Ensemble kinetic experiments

**Scheme 1.**
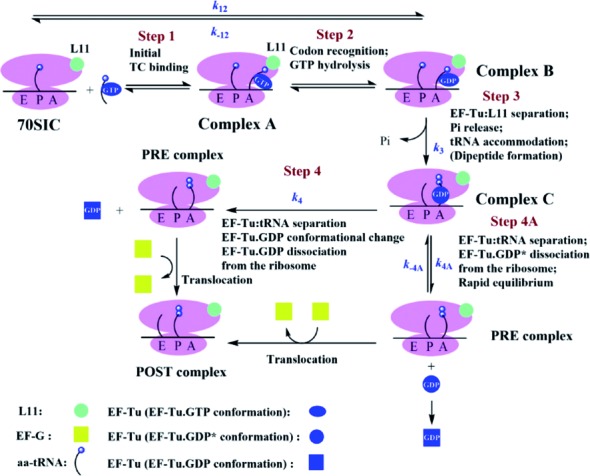
A simplified, quantitative model for TC interaction with 70SIC. Rate constants for different TC/70SIC combinations are presented in Table 2. Dipeptide formation is assumed to proceed very rapidly following tRNA accommodation (3). The metastable species EF-Tu·GDP* has not undergone the major conformational change associated with conversion of EF-Tu·GTP to EF-Tu·GDP.

Kinetic experiments were performed in buffer A at 25°C. The concentrations given below and in the figure legends are final after mixing. For P_i_ release experiments, GTP concentration was 50 μM, and no phosphoenolpyruvate and pyruvate kinase were added. TC solution was premixed with C-PBP [phosphate-binding protein labeled with 7-diethylamino-3-((((2-maleimidyl)ethyl) amino)carbonyl) coumarin] (2.5 μM), 7-methylguanosine (200 μM) and nucleoside phosphorylase (0.2 Unit/mL). Stopped-flow experiments were carried out on KinTek SF-300X stopped-flow spectrofluorometer. Cy3 was excited at 530 nm and monitored using either a 570 ± 10-nm band-pass filter for experiments employing three fluorescently labeled components or a 570 nm long-pass filter for all other experiments. Proflavin was excited at 462 nm and monitored using a 515 ± 15-nm long-pass filter. C-PBP was excited at 436 nm and monitored using a 455 nm long-pass filter. Apparent rate constants were obtained by exponential fitting using Origin (OriginLab). Global fittings of selected data sets to Scheme 1, generating rate constants reported in Table 2, were carried out using Scientist (MicroMath Research).

**Table 2.** Fitted rate constants for Scheme 1

	Reaction Components	*k*_12_, s^−1^ μM^−1^	*k*_-12_/*k*_12_, μM	*k*_3_, s^−1^	*k*_4_, s^−1 †^
1	TC^QSY9^ + 70SIC^Cy3^	36 ± 1	0.10 ± 0.02	15.4 ± 1.5	(19.6)^‡^
	Figure 1				
2	TC^QSY9/Cy3^ + 70SIC	100 ± 39	1.2 ± 0.1	19.9 ± 0.5	19.6 ± 1.1
	Figure 2				

^†^Because of uncertainty regarding the position of equilibrium in Step 4A under ensemble conditions, the simplifying assumption was made in carrying out the fitting that Complex C was dominant. The fitted value for *k*_4_ is thus a lower limit, but is likely to be only marginally lower than the true value.

^‡^Rate constant in parenthesis is fixed during fitting using Scientist.

### smFRET

smFRET studies were carried out at 21°C. 70S initiation complexes formed using a 5′-biotinylated mRNA (Dharmacon RNAi Tech.) were immobilized on a biotin/PEG-streptavidin coated glass surface (20). After washing away unbound complexes, collection of real-time fluorescence traces began 5 s prior to injecting 5 or 10 nM ternary complexes, which were preformed from EF-Tu, GTP and charged tRNAs. Recording continued for 60 s without further washing. An enzymatic deoxygenation system of 3 mg/ml glucose, 100 μg/ml glucose oxidase (Sigma-Aldrich), 40 μg/ml catalase (Roche) and 1.5 mM 6-hydroxy-2,5,7,8-tetramethyl-chromane-2-carboxylic acid (Trolox, Sigma-Aldrich—by dilution from a DMSO solution) was present in the final single-molecule imaging solutions to diminish fluorophore photobleaching and blinking. A custom-built objective-type total internal reflection fluorescence (TIRF) microscope was used to collect Cy3 and Cy5 (due to FRET from Cy3) fluorescence intensities on excitation by a 532 nm laser (16). The 11 ms integration time per frame was achieved by cutting the exposure area down to 128 pixels × 512 pixels without further binning. Other details of materials preparation, experimental setup, and data analysis were as previously described (16).

### FRET probability density plots

FRET probability density plots are two-dimensional contour maps plotted from time-resolved FRET histograms. For each plot, FRET traces were synchronized at the same specific event, such as the first or the last time points of FRET events. The FRET value is set to 0 when the Cy3 and Cy5 fluorescence signals were below 50% of the EF-Tu bound intensity.

### Synchronized FRET traces

Synchronized FRET traces are averaged traces from FRET traces synchronized at the same specific event, such as the first or the last time points of FRET events (21,22). Before averaging, for the pre-synchronization process, FRET traces were synchronized at the time points of FRET appearance. To obtain sets of FRET traces with constant duration, traces were extended after FRET disappearance using the FRET value of the last time point of each FRET event (21,22). Similarly, for the post-synchronization process, FRET traces were synchronized at the time points of FRET disappearance and were extended backward to before the appearance of FRET using the value of the first event time point.

## RESULTS

### Ensemble experiments based on the EF-Tu:L11 assay

**Figure 1.**
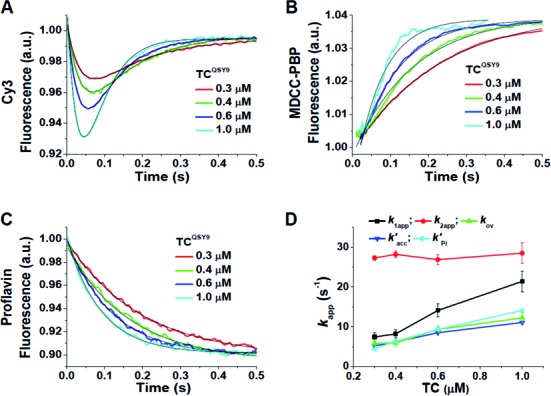
Ensemble studies of TC^QSY9^ reacting with 70SIC^Cy3^ (EF-Tu:L11 assay). (**A**)*–*(**C**) Changes of fluorescence on rapid mixing of 70SIC^Cy3^ (0.1 μM) with TC^QSY9^ as a function of TC^QSY9^ concentration. Lines through the traces are fit to Equation (1) (A) or Equation (2) (B) and (C). (A) Cy3 fluorescence. Experiments carried out either with 70SIC^Cy3^ containing fMet-tRNA^fMet^(prf) in the absence of C-PBP or with 70SIC^Cy3^ containing fMet-tRNA^fMet^ in the presence of C-PBP gave very similar results. Results presented are the average of both experiments. (B) C-PBP fluorescence, carried out using fMet-tRNA^fMet^. (C) fMet-tRNA^fMet^(prf) fluorescence, carried out in the absence of C-PBP. (**D**) *k*_app_ values derived from measurements of changes in the fluorescence of the label shown in parentheses, as presented in parts (A)*–*(C): Cy3, *k*_1app_ (black); Cy3, *k*_2app_ (red); Cy3, *k*_ov_ (green); prf (blue), *k’*_acc_; C-PBP (cyan), *k*’_Pi_.

Rapid mixing of TC^QSY9^ with a 70SIC^Cy3^ programmed with mRNA022 (19), which has a UUU codon following the AUG initiation codon, leads to a biphasic change in Cy3 fluorescence, with an initial decrease corresponding to TC binding to the ribosome that places EF-Tu position 348 in proximity to L11 position 87, followed by a restoration of fluorescence intensity as these two positions move apart (15). In Figure 1 we compare, all as a function of TC^QSY9^ concentration, rates of fluorescence change on rapid mixing of TC^QSY9^ and 70SIC^Cy3^ as measured by: (i) biphasic changes in Cy3 fluorescence (Figure 1A), (ii) increases in C-PBP fluorescence on binding to P_i_ released following EF-Tu·GTP hydrolysis (Figure 1B) (23) and (iii) decreases in P-site bound fMet-tRNA^fMet^(prf) fluorescence (Figure 1C). This latter change can be used to monitor accommodation of aa-tRNA into the A-site, since it proceeds at a rate identical to that of the fluorescence change of an A-site bound proflavin derivative of Phe-tRNA^Phe^ (see Supplemental Figure S1C in Ref. 24), that has been shown to signal accommodation into the A-site (3).

Fitting Equation (1) to the Cy3 fluorescence change demonstrates that *k*_1app_ increases linearly with TC^QSY9^ concentration whereas *k*_2app_ is independent of TC^QSY9^ concentration (Figure 1D), corresponding to a binding step and a following conformational rearrangement step, respectively. Fitting Equation (2) to both the increase in C-PBP fluorescence and the decrease in fMet-tRNA^fMet^(prf) fluorescence yields apparent first order constants denoted *k’*_Pi_ and *k’*_acc_,respectively. These latter constants, determined at several TC^QSY9^ concentrations, are in close agreement with each other and are approximately equal to the value of the overall rate constant for Cy3 fluorescence change, *k*_ov_, given by Equation (3) (Figure 1D). The reciprocals of *k*_ov_, *k’*_Pi_ and *k’*_acc_ provide estimates of the time it takes, following rapid mixing of ternary complex with 70S initiation complex, for EF-Tu to separate from L11, for P_i_ to be released and for aa-tRNA to be accommodated. The similarity in the values of these constants provides strong evidence that these three processes occur nearly simultaneously.

(1) \begin{equation*} {\rm F} = {\rm F}_0 + {\rm F}_1 {\rm e}^{{ - k}_{1{\rm app}^{\rm t} }} + {\rm F}_2 {\rm e}^{{ - k}_{2{\rm app}^{\rm t} }} \end{equation*}

(2) \begin{equation*} {\rm F} = {\rm F}_0 + {\rm F}_1 {\rm e}^{{ - k\prime}_{1{\rm app}^{\rm t} }} \end{equation*}

(3) \begin{equation*} k_{{\rm ov}} = k_{1{\rm app}} k_{2{\rm app}} /(k_{1{\rm app}} + k_{2{\rm app}} ) \end{equation*}

### Ensemble experiments based on the EF-Tu:tRNA assay

**Figure 2.**
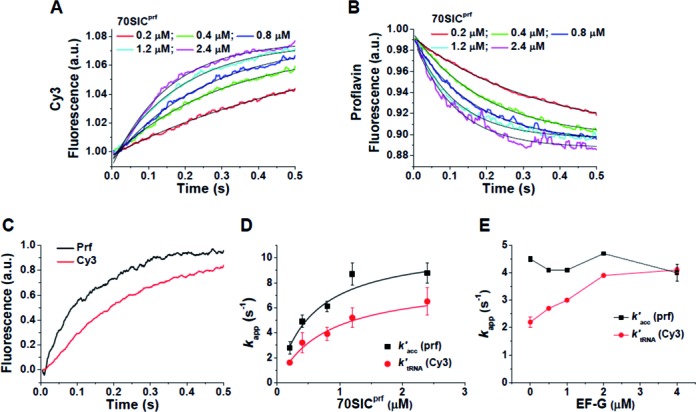
Ensemble studies of TC^QSY9/Cy3^ reaction with 70SIC (EF-Tu:tRNA assay). Changes of fluorescence on rapid mixing of 70SIC with TC^QSY9/Cy3^ (0.1 μM) as a function of 70SIC concentration. 70SIC was made up with fMet-tRNA^fMet^(prf). Each trace is an average of two independent determinations. (**A**) Cy3 fluorescence. (**B**) fMet-tRNA^fMet^(prf) fluorescence. In (A) and (B) lines through the traces are fit to Equation (2). (**C**) Representative sample data directly comparing the time-dependent progressions of prf and Cy3 fluorescence change in a common reaction mixture at 1.2 μM 70SIC. (**D**) *k’*_tRNA_(Cy3) and *k’*_acc_ (prf) as a function of 70SIC concentration, from parts (A) and (B). Solid lines are fits to the Michaelis-Menten function (Cy3: K_M_ 0.78 ± 0.14 μM, *V*_max_ 8.2 ± 0.9 s^−1^; prf: K_M_ 0.56 ± 0.16 μM, *V*_max_ 11.0 ± 1.3 s^−1^). (**E**) *k’*_tRNA_(Cy3) and *k’*_acc_ (prf) as a function of EF-G·GTP concentration. 70SIC, 0.4 μM.

In experiments paralleling those shown in Figure 1, the rate constant for the increase in Cy3-tRNA fluorescence on rapid mixing of TC^QSY9/Cy3^ with 70SIC made with fMet-tRNA^fMet^(prf), measuring EF-Tu separation from the D-loop of aa-tRNA, *k*’_tRNA_ (Figure 2A), and the rate constant for aa-tRNA accommodation, *k*’_acc_ (Figure 2B), measured as above (Figure 1C), were determined in successive injections into the stopped-flow mixer from a common reaction mixture. The prf fluorescence change proceeds more rapidly than the Cy3 fluorescence change, as shown by the representative sample data in Figure 2C. Fitting Equation (2) to each set of common results allowed for direct comparison of the apparent rate constants as a function of 70SIC concentration (Figure 2D), demonstrating that *k*’_acc_ is consistently higher than *k*’_tRNA_, by a factor of 1.6 ± 0.1 (Mean ± SEM; *P* < 0.001 in Student's *t*-test) measured in duplicate at five concentrations (Figure 2D). When fitted by Michaelis-Menten curves (solid curves in Figure 2D), the half saturation values are similar but the *V*_max_ values are significantly different (*P* < 0.02 by bootstrapping) (25). Since accommodation, which necessarily involves the release of the 3′-end of aa-tRNA from its binding to EF-Tu, occurs in concert with EF-Tu separation from L11 (Figure 1D), the difference in the rate constants presented in Figure 2C provides evidence that, as measured by ensemble reactions, ribosome-bound EF-Tu can separate from L11 and be released from its binding to the 3′-end of aa-tRNA (with rate constant *k*’_acc_) prior to its full separation from aa-tRNA (with rate constant *k*’_tRNA_). Because the fluorescent probes in TC^QSY9/Cy3^ are located within domain III of EF-Tu and the D-loop of tRNA, FRET efficiency is maintained between these two sites even as the 3′ acceptor end of tRNA dissociates from EF-Tu and accommodates into the 50S A-site. Importantly, simultaneous rapid mixing of TC^QSY9/Cy3^ together with EF-G·GTP, which catalyzes PRE complex conversion to POST complex, while having no effect on *k’*_acc_, increases the value of *k’*_tRNA_ (Figure 2E), abolishing the gap between the two at high EF-G·GTP concentration.

### Other labeling combinations

EF**-**Tu:L11 and EF-Tu:tRNA assays carried out with TC^Cy5^ and TC^Cy5/Cy3^ gave results that are kinetically indistinguishable from those carried out with with TC^QSY9^ and TC^QSY9/Cy3^, respectively (Supplementary Figure S2), demonstrating that Cy5 can replace QSY9 without loss of functional activity. On the other hand, replacement of TC^QSY9^ with TC^AV-Cy5^ or of TC^QSY9/Cy3^ with TC^AV-Cy5/Cy3^ slowed PRE complex formation by approximately 2-fold, as measured by the EF-Tu:L11 (Supplementary Figure S3A) or EF-Tu:tRNA (Supplementary Figure S3B) assays over wide concentration ranges (Supplementary Table S1). We attribute these rate decreases to replacement of the two Cys residues at positions 137 and 255 of EF-Tu with Ala and Val, respectively.

### smFRET studies of PRE complex formation

**Figure 3.**
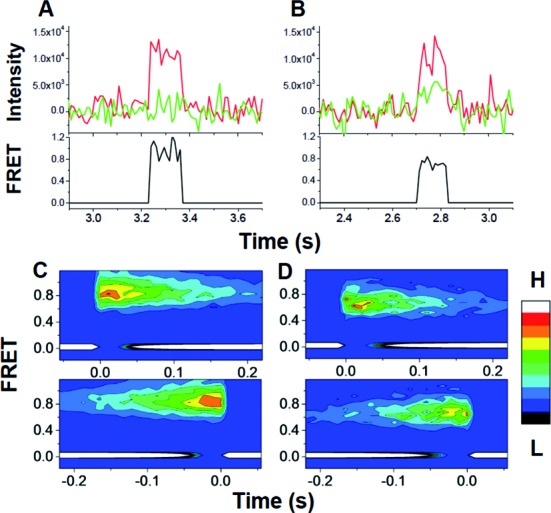
Real-time courses of single molecule fluorescence intensities and FRET during EF-Tu·GTP assisted tRNA delivery measured between (**A**) TC^Cy3^ and 70SIC^Cy5^ and (**B**) TC^Cy3/Cy5^ and 70SIC. TC (10 nM) was injected at time zero into the imaging chamber with immobilized 70SIC. (A) and (B) Cy3 (green) and Cy5 (red) fluorescence intensity traces under excitation of 532 nm TIRF illumination at 11 ms integration time per frame. The FRET ratio (black traces) was calculated as *I*_Cy5_/(*I*_Cy5_ + *I*_Cy3_). (**C**) and (**D**) FRET probability density plots for the reactions described in (A) and (B), respectively. Upper and lower traces are aligned to the first or last time points, respectively, of the FRET events as *t* = 0. Details of the procedure are described in Material and Methods. Besides the 0 FRET state, which corresponds to no fluorescence signal, only one FRET state is observed in each reaction. The same conclusion is reached from consideration of the synchronized FRET traces shown in Supplementary Figure S4.

**Figure 4.**
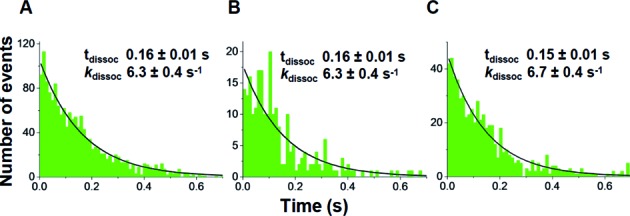
Dissociation times and rate constants for TC interaction with immobilized 70SIC, as determined by smFRET. (**A**) TC^Cy3^ (5 nM) and 70SIC^Cy5^; (**B**) TC^Cy3/Cy5^ (10 nM) and 70SIC; (**C**) TC^Cy3^ (10 nM) and 70SIC^Cy5^ in the presence of EF-G (4 μM) and 400 μM GTP. Lines are best fits to single exponentials, giving the indicated dissociation (*t*_dissoc_) times and corresponding values of *k*_dissoc_.

PRE complex formation using labeled TCs was further examined by single molecule FRET (smFRET), using a total internal reflection fluorescence (TIRF) microscope as described earlier (16). PRE complex formation was monitored by addition of either TC^Cy3^ or TC^Cy3/Cy5^ to immobilized 70SIC^Cy5^ (Figure 3A) or unlabeled 70SIC (Figure 3B), respectively. The results are very similar to one another, both showing constant FRET efficiencies [0.90 ± 0.01 (TC^Cy3^/70SIC^Cy5^); 0.69 ± 0.01 (^TCCy3/Cy5^)] over the entire duration of the FRET signal, as demonstrated by analysis of both FRET probability density plots (Figure 3C and D) and synchronized FRET traces (Supplementary Figure S4A and B) (21). During this time, the initially formed A/T complex is converted into the PRE complex. Given an *R*_o_ of 50–60 Å for the Cy3–Cy5 pair (26), such high efficiencies are consistent with the distances of 36–40 Å measured prior to accommodation between position 348 in EF-Tu and either position 87 in L11 or positions 16/17 in Phe-tRNA^Phe^ (Supplementary Figure S1), allowing for some variation in how the dyes are positioned in the two different experiments. The two experiments gave essentially identical values for *k*_dissoc_ (Figure 4A and B), measuring the rates of EF-Tu dissociation from the ribosome. Lifetimes of EF-Tu occupancy on the ribosome (*t*_dissoc_) averaging 150–160 ms are approximately 2-fold longer than expected from the ensemble rate constants reported in Table 2, which is likely due to the lower temperature employed (smFRET: 21ºC, ensemble: 25ºC). For the reaction of TC^Cy3^ and immobilized 70SIC^Cy5^, *k*_dissoc_ showed no significant change (Figure 4C) in the presence of a near-saturating value of EF-G·GTP (4 μM, *cf*. Figure 2D). Here again, only a single FRET state for ribosome-bound EF-Tu was observed (Supplementary Figure S4C). Thus, neither EF-Tu:L11 nor EF-Tu:tRNA FRET efficiency gives any indication of a major change in EF-Tu conformation while it is bound to the ribosome.

### A simplified quantitative model for TC interaction with 70SIC leading to PRE complex formation

The ensemble and smFRET measurements presented in Figures 1–4 allow formulation of a quantitative kinetic model for PRE complex formation (Scheme 1) that, while building on a more complete model presented earlier (3,27), stresses changes in EF-Tu conformation as well as in the interactions of EF-Tu with both the GAC (represented by L11) and aa-tRNA that occur during the process. Scheme 1 posits a minimum of three different conformational states for EF-Tu: EF-Tu·GTP, which corresponds to the conformation found in the isolated EF-Tu·GDPNP complex (8,9) that is essentially maintained when the TC is bound at the A/T site (28,29); EF-Tu·GDP*, an activated GTP-like conformation formed following GTP hydrolysis; and EF-Tu·GDP, which corresponds to the conformation found in the isolated EF-Tu·GDP complex (11,12).

The model identifies three complexes, A, B and C, as intermediates in PRE complex formation. Complex A corresponds to the initial complex rapidly formed between TC and the ribosome in Step 1 that is codon independent [Step 1 (3)]. Conversion of Complex A to Complex B requires codon recognition that is rapidly followed by GTP hydrolysis (30), grouped together as Step 2. An intermediate complex between A and B is formed when a non-hydrolyzable GTP analogue is substituted for GTP (15,28,29). In this intermediate complex, position 87 in L11 and the D-loop in aa-tRNA are each close enough to position 348 in EF-Tu (Supplementary Figure S1) to give rise to strong FRET signals, as measured either by quenching of Cy3 fluorescence (Figures 1A and 2A, Supplementary Figure S3A and C) or by increase in Cy5 fluorescence [Figure 3A and *B*, see also (15)]. Complex C is formed from Complex B by a multistep process (Step 3) that includes three substeps occurring nearly simultaneously: P_i_ release, aa-tRNA accommodation into the A-site, and EF-Tu separation from L11 (Figure 1D). This near simultaneity is in accord with earlier results showing similarity in the rates of P_i_ release and aa-tRNA accommodation (31), and, separately, aa-tRNA accommodation and EF-Tu separation from L11 (15). Complex C represents a branch point in the mechanism, from which EF-Tu·GDP* can either dissociate reversibly via Step 4A or, via Step 4, undergo a major conformational change to EF-Tu·GDP that proceeds essentially irreversibly either prior to or concomitantly with EF-Tu·GDP dissociation. Both branches obviously require complete EF-Tu separation from tRNA. The results presented in Figure 2D show that, in the absence of EF-G·GTP, such separation is not completed until after tRNA accommodation.

Globally fitting all of the kinetic data obtained with a given labeled TC to the kinetic mechanism shown in Scheme 1 allows evaluation of microscopic rate constants for the different steps in the model (Table 2). As the measurements reported in this paper do not resolve Step 1 from Step 2 (3), the overall conversion of 70SIC to Complex B is treated as a single step, characterized by forward and backward rate constants *k*_12_ and *k*_-12_, respectively. Rate constants for Steps 3 and 4 are characterized by rate constants *k*_3_ and *k*_4_, respectively_._ It is worth emphasizing that these microscopic rate constants differ from the apparent rate constants described above (*k’*_Pi_, *k’*_acc_, *k*_ov_, *k’*_tRNA_, Supplementary Table S1) which are derived simply by fitting the time dependence of each parameter change to Equations (1)–(3).

The fitted rate constants for the reaction of TC^QSY9^ with 70SIC^Cy3^ and of TC^QSY9/Cy3^ with 70SIC are compared in Table 2. Although Cy3 incorporation into Phe-tRNA^Phe^ in TC^QSY9/Cy3^ significantly weakens Complex B formation, mostly via increasing *k*_-12_, it has little effect on the rate of Complex B conversion to Complex C, as measured by *k*_3_ (Table 2, row 1 versus row 2). Similarly, *k*_3_ has essentially the same value for reaction of TC^AV-Cy5^ with 70SIC^Cy3^ and of TC^AV-Cy5/Cy3^ with 70SIC, but Complex B formation is weakened in the latter case (Supplementary Table S2). These effects on Complex B formation are consistent with other studies of ours showing somewhat reduced activity in protein synthesis of Cy3-labeled versus unlabeled Phe-tRNA^Phe^ under conditions of limiting Phe-tRNA^Phe^ concentration (32).

## DISCUSSION

Here we apply two recently developed FRET assays monitoring EF-Tu distances to aminoacyl-tRNA and to protein L11 in the GAC (15) to formulate a detailed kinetic mechanism for EF-Tu interaction with the ribosome and with tRNA during PRE complex formation (Scheme 1). According to this mechanism, the three substeps occurring nearly simultaneously in Step 3, P_i_ release, aa-tRNA accommodation into the A-site, and EF-Tu separation from L11, precede EF-Tu dissociation from the ribosome. In addition, EF-Tu dissociation from the ribosome either precedes (Step 4A), or occurs concomitantly with (Step 4), formation of what we have designated as the ‘EF-Tu·GDP conformation’. In contrast, it has been proposed that EF-Tu dissociation precedes accommodation (33) and that the EF-Tu·GDP conformation is formed on the ribosome prior to its dissociation (33,34), but compelling evidence has not been presented supporting either of these proposals.

In this paper we present direct kinetic evidence that accommodation precedes complete EF-Tu separation from tRNA, based on the observation that *k*’_acc_ is larger than *k*’_tRNA_ (Figures 2C and 2D). It follows from this observation that the 3′-end of aa-tRNA must migrate from its binding site within EF-Tu while it is in the A/T site [ndb 4V5L (29)], to the A-site on the 50S subunit [ndb 4V5D (35)], a distance of some 80 Å, prior to EF-Tu dissociation from the ribosome. A partial precedent for such a mechanism is the rapid translocation of the 3′-end of aa-tRNA between the activation and editing sites of aminoacyl-tRNA synthetases (aaRSs) during proofreading (36), which occurs prior to dissociation of the aa-tRNA.RS complex. The distance between these two aaRS sites is ∼ 35 Å (37), so that rapid movement between them is achievable as a consequence of the flexibility of the single-stranded nucleotides at the 3′-end of aa-tRNA. The much larger displacement for movement from the A/T site to the A-site suggests that, in addition to movement of the 3′-end, a further structural rearrangement is required that, while maintaining the distance between the tRNA D-loop and residue 348 in EF-Tu, rotates aa-tRNA into the fully accommodated position, allowing peptide-bond formation to take place. We speculate that this rearrangement, which may include the straightening of the tRNA anticodon stem of the tRNA as it leaves the A/T site (38), is induced by the change in the bound EF-Tu structure as it converts from the EF-Tu·GTP to the EF-Tu·GDP* conformation (Scheme 1). We further speculate, as indicated schematically in Scheme 1 and consistent with the loss of FRET interaction with L11 (Figure 1A), that as a result of this change, EF-Tu moves from a position embedded within the ribosome to one in which its contacts with the ribosome are largely confined to its domain 3 interaction with the acceptor stem of aa-tRNA, in a manner which allows the 3′-end to participate in peptide bond formation.

When does the EF-Tu·GDP conformation form? Direct evidence that EF-Tu can successfully deliver cognate aa-tRNA to and dissociate from the ribosome without undergoing such major conformational change is presented in a related single molecule study by our group, measuring intramolecular FRET efficiency in an EF-Tu doubly labeled with donor and acceptor probes (Kavaliauskus et al., submitted for publication). The notion that EF-Tu·GDP* formed on the ribosome could persist at least transiently following dissociation is supported by considerable evidence that GTPases like EF-Tu fluctuate in a dynamic equilibrium between different conformational states, independently of whether GTP or GDP is bound (39). Two striking examples of this phenomenon are the structures of the GDP complexes of bacterial selenocysteine specific elongation factor SelB (40) and a Rab6A’ mutant (41), both of which are in GTP-like conformations.

The evidence presented in this paper supporting this view, along with the inclusion of Complex C as a branch point in Scheme 1, is more inferential in nature, and derives from our efforts to explain two observations that appear puzzling at first glance. The first is that smFRET experiments show only a single, high L11:EF-Tu FRET value (Supplementary Figure S3A), whereas ensemble experiments, showing that ribosome-bound EF-Tu preferentially separates from L11 prior to its complete release from aa-tRNA, suggest that a low EF-Tu:L11 FRET state, corresponding to Complex C, should also be occupied before dissociation. The most straightforward explanation of this seeming disparity is the difference in reactant concentrations employed in our single molecule and ensemble experiments. The ensemble experiments employ relatively high concentrations of ribosomes and TC, such that Step 4A is rapidly reversible and favors occupancy of Complex C. This allows Complex C to accumulate transiently, since it is only removed by irreversible formation of PRE complex via Step 4. In contrast, because single molecule experiments are performed at very low concentrations of ribosomes and TC, dissociation of EF-Tu·GDP* via Step 4A is effectively irreversible. Thus, in the smFRET experiments, EF-Tu dissociation proceeds irreversibly via both Steps 4A and 4, limiting the transient buildup of Complex C to below the level of detection. The absence of detectable Complex C in the smFRET experiments indicates that its lifetime must be ≤10–20 ms, given the 11 ms integration time per frame used in the experiment. A consequence of these values is that *k*_4A_ is ≥ *k*_4_.

The second observation is that EF-G·GTP increases the rate of complete EF-Tu:tRNA separation, as measured in ensemble experiments (Figure 2E), while having no effect on the dwell time of the ribosome-bound EF-Tu:tRNA FRET signal measured by smFRET (Figure 4C, Supplementary Figure S4). According to Scheme 1, added EF-G·GTP at high concentration can compete with EF-Tu.GDP* for binding to the PRE complex, leading to translocation. Such competition renders Step 4A essentially irreversible, so that, even under ensemble conditions, Complex C does not accumulate, and the rate of complete EF-Tu:tRNA separation is limited mainly by Complex B conversion to Complex C, i.e., it proceeds at a rate comparable to that of EF-Tu separation from L11, P_i_ release or aa-tRNA accommodation (Figures 1D and 2E). The fact that added EF-G·GTP has no effect on the dwell time of the ribosome-bound EF-Tu:tRNA (Figure 4C) is evidence against an alternative explanation that EF-G·GTP stimulates complete EF-Tu:tRNA separation by an allosteric mechanism. Interestingly, given the high cellular concentration of EF-G [∼20 μM, stoichiometrically equivalent to ribosomes (42)], Step 4A may be the major route for EF-Tu dissociation *in vivo*.

In summary, we describe the dynamics of EF-Tu interaction with tRNA and with the ribosome during pretranslocation complex formation by combining the results of stopped-flow ensemble and single-molecule FRET measurements. We demonstrate that EF-Tu separation from L11 within the GTPase Associated Center of the ribosome can occur prior to EF-Tu separation from the D-loop of Phe-tRNA^Phe^, which may happen at a late stage of accommodation. Furthermore, our results strongly suggest that EF-Tu·GDP dissociates from the ribosome via two different pathways, one more rapid and reversible and one slower and essentially irreversible, that may correspond to two different EF-Tu·GDP conformations on the ribosome.

## Supplementary Material

SUPPLEMENTARY DATA
