# Supplementary material for: EF-Tu dynamics during pre-translocation complex formation: EF-Tu·GDP exits the ribosome via two different pathways
Source: Nucleic Acids Res. 2015 Sep 3;43(19):9519–28. doi: 10.1093/nar/gkv856 (PMC4627077; doi:10.1093/nar/gkv856)
Supplement: SUPPLEMENTARY DATA [file supp_43_19_9519__index.html]

EF-Tu dynamics during pre-translocation complex formation: EF-Tu·GDP exits the ribosome via two different pathways — SUPPLEMENTARY DATA 

# EF-Tu dynamics during pre-translocation complex formation: EF-Tu·GDP exits the ribosome via two different pathways

## SUPPLEMENTARY DATA

- SUPPLEMENTARY DATA
