# Supplementary material for: EF-Tu dynamics during pre-translocation complex formation: EF-Tu·GDP exits the ribosome via two different pathways
Source: Nucleic Acids Res. 2015 Sep 3;43(19):9519–28. doi: 10.1093/nar/gkv856 (PMC4627077; doi:10.1093/nar/gkv856)
Supplement: SUPPLEMENTARY DATA [file supp_gkv856_nar-01790-v-2015-File003.pdf]

Supporting Information

EF-Tu Dynamics During Pre-translocation Complex Formation: EF-Tu·GDP exits the ribosome via two different pathways.

Wei Liu[1], Chunlai Chen[2], Darius Kavaliauskas[3], Charlotte R. Knudsen[3], Yale E. Goldman[2] and Barry S. Cooperman[1,*]

[1] Department of Chemistry, University of Pennsylvania, Philadelphia, PA 19104
[2] Pennsylvania Muscle Institute, School of Medicine, University of Pennsylvania, Philadelphia, PA 19104
[3] Department of Molecular Biology and Genetics and Interdisciplinary Nanoscience Center (iNANO), Aarhus University, DK-8000 Aarhus C, Denmark

* To whom correspondence should be addressed. Tel: +1 215 898 6330; E-mail: coopman@pobox.upenn.edu

Present address: [Wei Liu], Pfizer, Inc, 610 Main St., Cambridge, MA 02139; [Chunlai Chen], School of Life Sciences, Tsinghua University, Beijing,China,100084; [Darius Kavaliauskas] Thermo Fisher Scientific Baltics, LT-02241 Vilnius, Lithuania.

Table S1. Apparent rate constants as a function of EF-Tu variant and dye labeling

Tu-L11 assay, k_{ov}, s^{-1} Equation 3	TC, μM	0.2	0.3	0.4	0.6	0.8	1.0
	$70SIC^{Cy3}$ + TC^{QSY9}		5.9 ± 0.7	5.9 ± 0.8	9.3 ± 0.8	-	12.2 ± 1.3
	$70SIC^{Cy3}$ + $TC^{AV\text{-}Cy5}$	1.8 ± 0.2		2.8 ± 0.2	4.0 ± 0.5	4.9 ± 0.6	5.7 ± 0.5
Tu-tRNA assay, k'_{tRNA}, s^{-1} Equation 2	70SIC, μM		0.4	0.8	1.2	2.0	2.4
	70SIC + $TC^{QSY9/Cy3}$		3.2 ± 0.8	3.9 ± 0.5	5.2 ± 0.8	-	6.5 ± 1.1
	70SIC + $TC^{AV\text{-}Cy5/Cy3}$		1.8	2.5	2.7	3.5	-

Table S2. Fitted rate constants for Scheme 1

**Reaction Components**	k_{12}, s^{-1} μM^{-1}	k_{-12}/k_{12}, μM	k_3, s^{-1}	k_4, s^{-1} †
$TC^{AV\text{-}Cy5}$ + $70SIC^{Cy3}$ Figure S3A and C	5.5 ± 0.4	≤ 0.3	27 ± 2	(5.6)‡
$TC^{AV\text{-}Cy5/Cy3}$ + 70SIC Figure S3B and D	5.5 ± 1.1	≤ 1.0	27 ± 8	5.6 ± 0.6

†Because of uncertainty regarding the position of equilibrium in Step 4A under ensemble conditions, the simplifying assumption was made in carrying out the fitting that Complex C was dominant. The fitted value for k_4 is thus a lower limit, but is likely to be only marginally lower than the true value.
‡Rate constants in parentheses are fixed during fitting using Scientist.

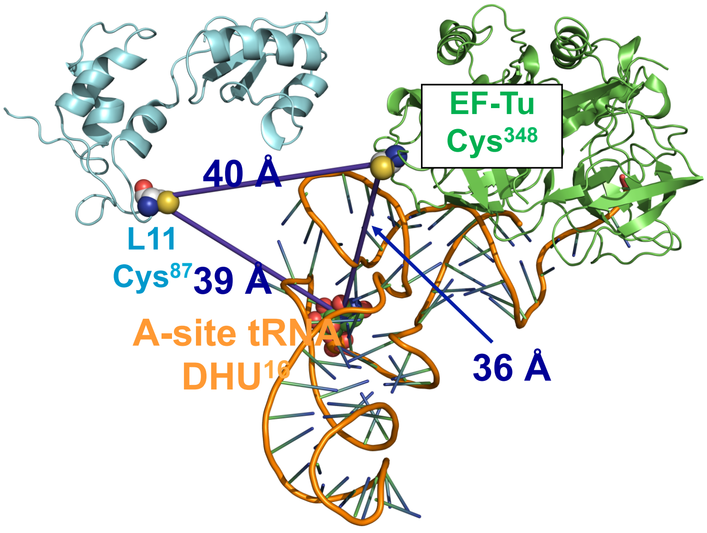

Figure S1. Distances between residue numbers 348 (C_a, EF-Tu), 87 (C_a, L11) and 16 (N-1, aminoacyl-tRNA) in a kirromycin- and paromomycin- stabilized ribosome complex with Thr-$tRNA^{Thr}$ bound in the A/T site. Protein Data Bank accession codes 2WRN, 2WRO, 2WRQ, and 2WRR.

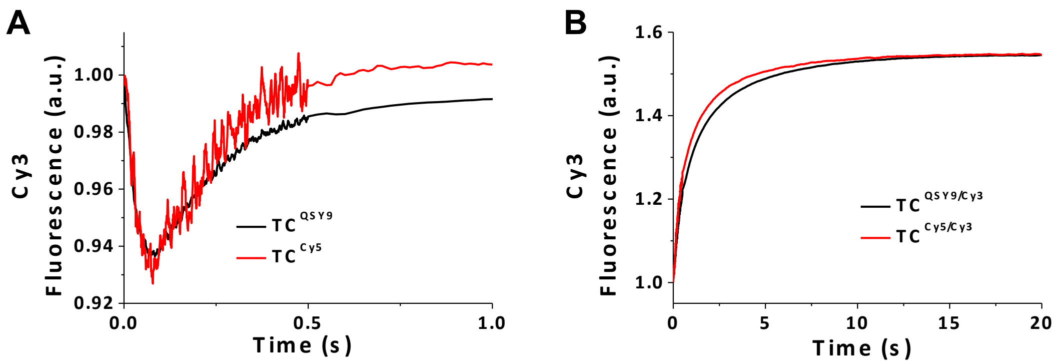

Figure S2. QSY9 vs. Cy5 labeling of EF-Tu. Comparisons of rates of reaction of (**A**) TC^{QSY9} or TC^{Cy5} (0.4 μM) with 70SIC^{Cy3} (0.1 μM), and (**B**) TC$^{QSY9/Cy3}$ or TC$^{Cy5/Cy3}$ (0.1 μM) with 70SIC (0.4 μM).

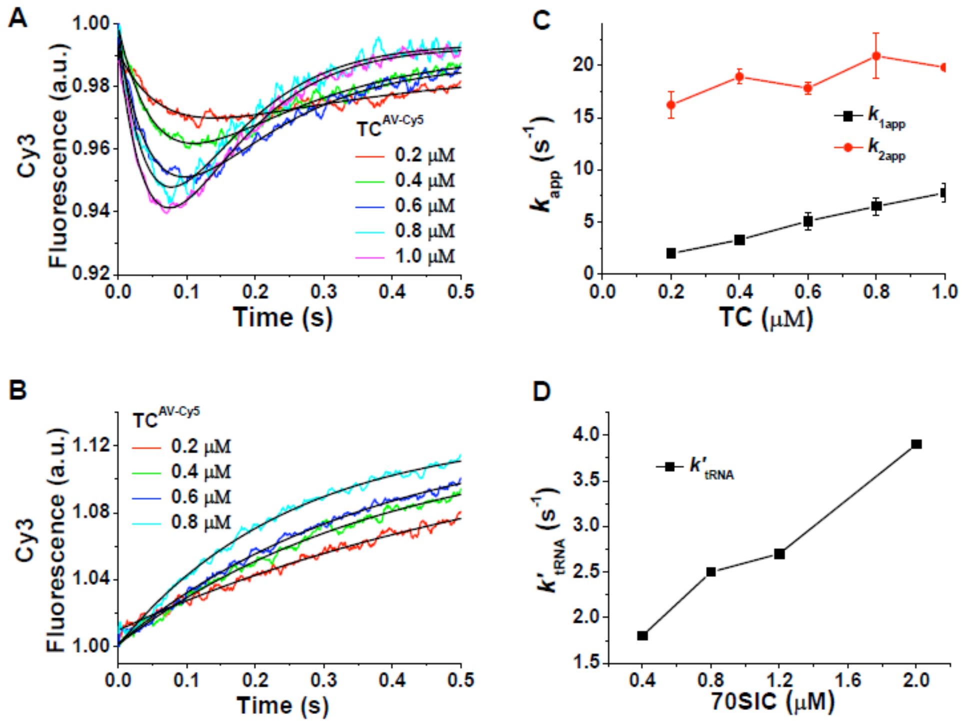

Figure S3. Ensemble studies using EF-Tu$^{AV\text{-}Cy5}$. (**A**) and (**B**) Changes of Cy3 fluorescence on rapid mixing of: (**A**) 70SIC^{Cy3} (0.1 μM) with various concentrations of TC$^{AV\text{-}Cy5}$; (**B**) TC$^{AV\text{-}Cy5/Cy3}$ (0.1 μM) with various concentrations of 70SIC. Lines through the traces in parts (**A**) and (**B**) are fit to Equations 1 and 2, respectively. (**C**) Plots of k_{1app} and k_{2app} for the curves shown in (**A**). (**D**) Plot of k'_{tRNA} for the curves shown in (**B**).

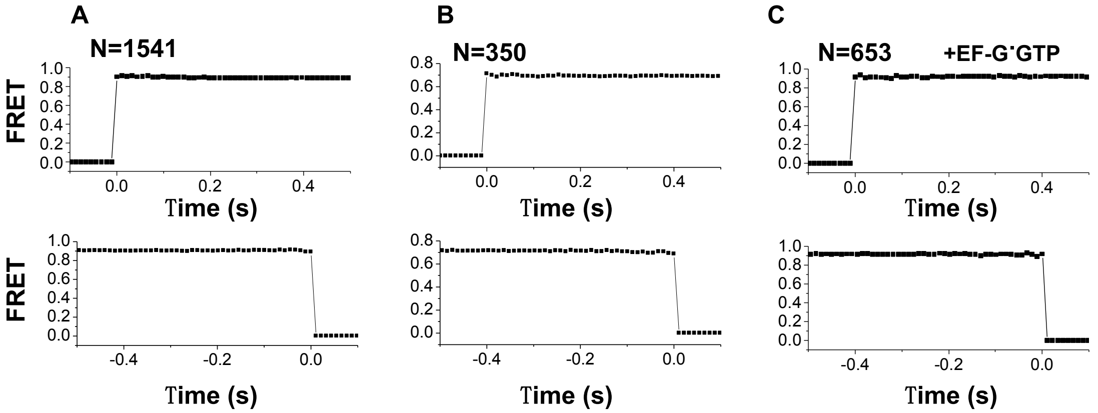

Figure S4. Synchronized smFRET values. (**A**) For TC^{Cy3} added to $70SIC^{Cy5}$. (**B**) For $TC^{Cy3/Cy5}$ added to 70SIC. (**C**) For TC^{Cy3} added to $70SIC^{Cy5}$ in the presence of 4 μM EF-G·GTP. Upper and lower plots are synchronized to FRET appearance and disappearance, respectively.
